# Gastronomic paradigm shifts revisited: from culinary abstraction to a post-digital integrative cuisine

**DOI:** 10.3389/fnut.2025.1649945

**Published:** 2025-11-20

**Authors:** Raimundo G. del Moral

**Affiliations:** Department of Pathological Anatomy and History of Science, University of Granada, and Institute of Biosanitary Research of Granada (ibs.GRANADA), Granada, Spain

**Keywords:** gastronomic paradigms, post-digital gastronomy, regenerative cuisine, biotechnological food design, multispecies ethics, artificial intelligence in food, ecological innovation, hybrid knowledge systems

## Abstract

This article proposes the existence of a fifth gastronomic paradigm, which we term *Post-Digital Integrative Cuisine*, marking an epistemological rupture in the evolution of Western haute cuisine. Drawing on Kuhnian theory, regenerative ethics, and interdisciplinary case studies, we argue that this paradigm is defined not by stylistic or technical novelty, but by its reconfiguration of gastronomy as a multispecies, systemic, and anticipatory practice. This model integrates microbial fermentation, biotechnological innovation, ancestral knowledge, and artificial intelligence into culinary design. It prioritizes ecological regeneration, distributed authorship, and epistemic pluralism over individual genius or sensory spectacle. Empirical examples illustrate how food becomes a medium for world-making, narrative design, and regenerative aesthetics. We analyze how this paradigm realigns culinary aesthetics with ethical and ecological values through principles such as circular gastronomy, fermentation as epistemology, dematerialized luxury, and AI-assisted creativity. At the same time, we critically assess persistent tensions: the risk of elitism, techno-solutionism, and symbolic commodification. Key contributions include (1) a Kuhnian mapping of gastronomic paradigms, (2) a conceptual definition of the fifth paradigm's post-disciplinary logic, and (3) an exploration of anticipatory imagination and food as a speculative design tool. By situating gastronomy within the frameworks of environmental humanities, futures studies, and hybrid knowledge systems, this article reframes haute cuisine as a cultural technology for ecological care, multispecies justice, and epistemic transformation. The Post-Digital Integrative Cuisine is not a utopian projection but a distributed present—demanding visionary courage, ethical coherence, and systemic participation.

## 1 Introduction

A previous study adopted a Kuhnian framework to interpret the evolution of Western haute cuisine since the French Revolution, identifying four foundational paradigms that have shaped its historical and epistemological development (1).

In summary, these four gastronomic paradigms allow us to map the conceptual transformation experienced by Western haute cuisine from that time to the present day (1). While La Varenne laid the foundations for systematized culinary thought a century earlier (2), it was Carême who, after the French Revolution and through the First Gastronomic Paradigm, democratized aristocratic cuisine by bringing culinary art into the public sphere, also creating the contemporary restaurant model that persists to this day. The Second Paradigm, which was consolidated in the mid-19th century, was articulated by Escoffier around the rationalization and codification of culinary knowledge, the hierarchical organization of modern cuisine, and the complete standardization of techniques and recipes. The Third Paradigm, which emerged in the mid-20th century, was represented by Nouvelle Cuisine, which prioritized dishes based on creativity, lightness, and the individual authorship of the chef. Finally, the Fourth Paradigm, known as Culinary Abstraction, developed between the late 20th and early 21st centuries, focusing on the sensory manipulation of the diner through food texture, aesthetic innovation, and the conceptual deconstruction of dishes, as exemplified by the abstract cuisine of Ferran Adrià (1).

Currently, gastronomy is undergoing a new epistemological rupture. As Perullo has pointed out, this rupture entails a redefinition of smell, taste, and touch, which are no longer primarily visual, memorized, and codified experiences and become a multisensory, embodied, and ecological phenomenon, anchored in forms of relational knowledge and haptic perception (3).

Just a few years ago, gastronomy and haute cuisine were already showing signs of transformation and conceptual uncertainty, although it was unclear whether such developments constituted a paradigm shift or simply a chaotic phase of transition. Given the changes since the COVID-19 pandemic, and based on evidence and analysis, it is possible to rethink that initial hypothesis. In my opinion, the period once interpreted as a possible fifth paradigm should be better understood as a Kuhnian crisis, that is, as the phase of instability that emerged after the exhaustion of Culinary Abstraction symbolized by the closure of the ElBulli restaurant in Roses (Girona, Spain). This chaotic period has been characterized by the fragmentation of the dominant type of cuisine, the proliferation of aesthetic relativism, and the symbolic—often superficial—adoption of sustainability discourses, without the emergence of a new coherent culinary epistemology capable of replacing the system once exemplified by Ferran Adrià's abstract cuisine. This symbolic fragmentation has also been reflected in the growing mediation of the culinary experience by digital narratives and affective branding strategies, a phenomenon especially visible in gastronomic practices linked to tourism (4).

According to Kuhn's theoretical model (5), a new paradigm is consolidated when it manages to resolve previous anomalies, reorient the fundamental purposes of a field of knowledge and obtain broad recognition within its community. Historically, these paradigmatic shifts have been catalyzed by two main vectors: disruptive technological developments and profound social transformations (6–8).

As several recent studies suggest, these vectors have intensified notably in recent years, particularly following the COVID-19 pandemic (9). Consequently, contemporary gastronomy increasingly aligns with the conditions Kuhn described for the emergence of a new paradigm. This includes epistemic disruption, technological innovation, and sociocultural reorientation.

In this article, I argue that the fifth paradigm of contemporary haute cuisine, rather than being in a chaotic phase of transition, is becoming clearly visible. This fact is defined by the convergence of regenerative ethics in food procurement and development — understood here as a normative framework centered on ecological repair, multispecies interdependence, and long-term systemic care —, biotechnological innovation in food production and preparation, and the emergence of digital and post-digital knowledge applied to cooking. Recent advances in computational gastronomy, such as data-driven recipe generation, flavor prediction, and cross-cultural modeling, illustrate how artificial intelligence is transforming the structure of culinary knowledge (10). Unlike previous moments of rupture—such as the arrival of Nouvelle Cuisine or Culinary Abstraction—often driven by stylistic or technical novelties, the current transformation reflects a profound redefinition of the role of gastronomy in an ecologically threatened, socially complex, and technologically hybrid world. The emerging paradigm proposed in this article realigns culinary practice with planetary survival, systemic health, and collective intelligence, signaling a systemic reorientation of gastronomic excellence grounded in ethical coherence and transformative praxis. Therefore, it marks not only a culinary innovation, but a paradigmatic reconfiguration of gastronomy as a cultural, ecological, and epistemic force. Table 1 offers a comparative overview of the five paradigms proposed, from public cuisine to post-digital regeneration.

**Table 1:** Chronology of gastronomic paradigms (17th−21st century).

**Paradigm**	**Key figures**	**Period**	**Epistemic focus**	**Aesthetic features**
I. Public Cuisine	Carême	Early 19th c.	Public codification, aristocratic model	Opulence, public display
II. Rational Codification	Escoffier	Mid–late 19th c.	Systematization, technique hierarchy	Uniformity, structure
III. Nouvelle Cuisine	Point, Bocuse	1960s−1980s	Individual authorship, lightness	Creativity, freshness
IV. Culinary Abstraction	Adrià, Blumenthal	1990s−2010s	Conceptual deconstruction, affect	Surprise, dematerialization
V. Post-Digital Integrative Cuisine	Redzepi, Aduriz, Crenn	2020s–present	Regeneration, multispecies logic	Narrative, systemic aesthetics

Chronological overview of the five main gastronomic paradigms from the 19th to the 21st century, highlighting their epistemic foundations and aesthetic orientations. This framework situates the fifth paradigm—Post-Digital Integrative Cuisine—within a historical continuum of conceptual ruptures.

The post-ElBulli period, often framed as an age of aesthetic excess or creative exhaustion, exposed a deeper epistemological crisis: the collapse of a shared horizon of meaning in contemporary gastronomy (1). The fragmentation of conceptual models, the proliferation of unaffiliated techniques, and the market-driven pursuit of novelty without a coherent framework led to what could be described as paradigm fatigue. The Fifth Paradigm, as proposed here, does not simply introduce new styles or tools—it responds structurally to this fragmentation by offering a systemic realignment. It reintegrates ethics, aesthetics, epistemology, and material practice through a post-disciplinary approach that values hybrid knowledge, multispecies co-authorship, and anticipatory imagination. In this sense, it marks not a rupture for its own sake, but a reparative act: a reweaving of gastronomy's symbolic, ecological, and epistemic functions after a phase of disarticulation.

## 2 Conceptual delimitation: distinguishing gastronomic paradigms from trends and movements

A gastronomic paradigm shift, in the sense used here, goes beyond a mere shift toward more ethical or sustainable forms of eating. As Fischler points out, gastronomy has always sought to transform nature into culture through the use of symbols, sensory sophistication, and the aesthetic interpretation of food (11). Therefore, food proposals centered exclusively on health, ethics, sustainability, or identity politics need to be elevated through culinary authorship, sensory refinement, and gastronomic pleasure within public spaces such as restaurants or equivalent spaces. Furthermore, while veganism and other food movements can be culturally significant, they rarely meet the requirements necessary to propose the emergence of a new gastronomic paradigm unless they are mediated by techniques, aesthetics, and restaurants that generate new culinary knowledge. For example, DaSilva et al. (12) describe how some forms of veganism function as serious leisure alternatives in response to current social needs, but remain primarily identity-based practices, not gastronomic innovations per se.

Similarly, both ethnic cuisines and contemporary fusion cuisine are often framed as a global innovation in each territory where they are established. However, as Spence points out, their widespread appeal often stems from commercial viability, exoticism, or the reinforcement of identity narratives, rather than to epistemological rupture or systemic culinary reconfiguration (13). As such, fusion remains an aesthetic phenomenon shaped by cultural exchange, not a paradigm shift in the Kuhnian sense.

Even within the rhetoric of innovation, many emerging culinary trends rely more on lifestyle branding than on genuine epistemic novelty. As several studies on Western perceptions of ethnic cuisines suggest, much of the perceived innovation stems more from market positioning and exotic appeal than from the development of new gastronomic logics (14). Similarly, recent analyses of future food trends emphasize the need to distinguish between mere technological enhancement and the emergence of a true paradigm shift. While future cuisines are likely to incorporate tools such as artificial intelligence, plant-based proteins, and territorial sustainability frameworks, these elements will only acquire paradigmatic significance when embedded within a coherent and self-sustaining system of knowledge, aesthetics, and symbolic meaning (7).

Accordingly, under strict paradigmatic criteria, fusion cuisine—however culturally expressive or commercially successful—cannot be considered part of the fifth gastronomic paradigm proposed in this article.

## 3 A fifth paradigm: post-digital integrative cuisine

We propose the term Postdigital Integrative Cuisine to define the fifth gastronomic paradigm, which marks a decisive epistemological break with the preceding era of Culinary Abstraction. This term restricts the paradigm to the systemic interdependence between cooks and their environment, ecological regeneration and sustainability, the dissolution of the boundaries between traditional and synthetic ingredients, the progressive incorporation of microbial agents into the cutting-edge culinary recipes, and the incorporation of digital intelligence and robotics into gastronomy.

The term integrative underscores the interdisciplinary and co-creative nature of this culinary model, where chefs, scientists, artisans, AI, microbes, and local agronomic communities collaborate to construct new culinary formulas. It reflects the convergence of ancestral techniques, precision fermentation, synthetic and/or sustainable ecology, and narrative design within a unified framework of ethical, aesthetic, and material transformation. Meanwhile, the post-digital era signals the maturity of algorithmic systems as integrated, often invisible, agents in gastronomic innovation; not as spectacle, but as tools for regenerating landscapes, reprogramming fermentation ecology, and expanding cultural imaginaries. In this paradigm, cooking is no longer simply a craft or artistic expression, but an epistemological act that actively participates in shaping humanity's ecological future and a multispecies ethic.

The Fifth Paradigm must be distinguished not only from previous paradigms, but also from transient trends that emerge in response to media visibility or commercial differentiation. Unlike such trends, which often privilege novelty for branding purposes, the Fifth Paradigm articulates a coherent, systemic logic grounded in epistemological realignment. Its validity does not rely on aesthetic markers or individual chefs, but on its capacity to integrate hybrid knowledge systems, to reconfigure culinary authorship, and to foster long-term regenerative thinking. This makes it generalizable beyond any single institution or chef's persona. Projects such as Midunu (Africa), Owamni (North America), and the work of the Alchemist Lab (Europe) or the research-driven practice of Andoni Luis Aduriz at Mugaritz (Spain) demonstrate how post-digital gastronomy can function as a research platform, not merely a performance or spectacle. The distinction lies in whether culinary innovation generates knowledge with structural impact—or merely temporary distinction.

### 3.1 Radical ethics and food biotechnologies

#### 3.1.1 Cultured meats

The fifth gastronomic paradigm places radical ethics at the center of culinary innovation, transforming both the materiality of food and the moral framework that guides gastronomic practices. Cultured meat technologies represent one of the most significant advances in this field. By cultivating animal muscle tissue *in vitro*, this biotechnology seeks to decouple meat production from traditional livestock farming, thereby addressing ethical concerns related to animal welfare, environmental degradation, and food safety (15). However, the transition of cultured meat from the laboratory to the market requires not only technical innovation but also the establishment of robust regulatory frameworks and strategies to foster public trust.

The conceptual evolution of cultured meat must move from representing a technological and scientific novelty to being a plausible strategy for sustainable food systems. The appropriate epistemological shift is necessary for consumers and institutions to firmly believe that the development of alternatives to conventional meat can replace it, not only in terms of sensory imitation, but also because of their stronger ethical and ecological legitimacy (16).

In addition to ethical concerns, food safety has become a critical research priority in cultured meat technology. Ensuring its microbiological safety, nutritional suitability, and long-term stability requires extensive research into new culture media, support materials, and production processes (17).

Furthermore, debates surrounding the “naturalness” of cultured meat persist. While some critics argue that *in vitro* meat is inherently artificial and therefore objectionable, others maintain that its ability to minimize harm to animals and ecosystems outweighs such concerns (18). As Welin argues, concerns about the “naturalness” of cultured meat must be reconsidered, recognizing that it offers tangible improvements in animal welfare and environmental stewardship (18). And this is one of the great challenges facing the chefs of the future: replacing those wonderful meats from older beef, suckling lamb, or game, now available to very few diners and at astronomical prices, with a new, sustainable, and inexhaustible meat, with a sensorially gratifying texture and flavor.

Beyond utilitarian, sustainability, and deontological frameworks, virtue ethics introduces a more introspective critique. Alvaro (19) suggests that even the motivation behind lab-grown meat reflects a less-than-virtuous human disposition, one that prioritizes sensory gratification over the cultivation of temperance and a healthy relationship with food. From this highly debatable perspective, the pursuit of synthetically recreated meat—despite the growing availability of plant-based alternatives, many of which still struggle to match the textural fidelity, umami depth, and cultural embeddedness of traditional meat—would signal a disregard for virtues such as moderation and ecological humility, which underlie mainstream vegetarian and vegan ethics. In this regard, Lima et al. (20) analyze the rapid development of alternative protein sources, including plant-based, insect-based, and mycoprotein products, alongside those grown in the laboratory. These innovations aim to replicate the nutritional and sensory properties of animal-based foods while addressing ethical, environmental, and health concerns. However, significant challenges remain regarding consumer acceptance, affordability, nutritional optimization, and technological scalability. It can therefore be concluded that the development of alternative proteins reflects not only technological progress, but also a broader ethical and ecological redefinition of how food systems might evolve in line with post-anthropocentric values.

#### 3.1.2 Biofermentations

Along with the development of cultured meat, fermentation is experiencing a renaissance in contemporary gastronomy, emerging not only as a traditional practice but as a cornerstone of culinary innovation. Chefs, scientists, and artisans are collaborating to develop new microbial strategies that enhance the nutritional properties of foods, optimize food safety, reduce waste, and recover flavor profiles often absent from contemporary haute cuisine (21).

In this context, fermentation is no longer conceived as a passive, often unwanted, biochemical process in food, but as a creative and symbolic act that revitalizes ancestral knowledge. Fermentation repositions microbes as active co-agents in culinary transformation, aligning gastronomy with microbiome ecology, multispecies interaction, and post-anthropocentric ethics (22). Current innovations include controlled fermentations that generate new textures and molecules rich in umami or with flavors similar to those of cheese, as well as specific microbial consortia designed to enhance probiotic functionality and nutrient bioavailability. At the same time, wild and spontaneous fermentations are increasingly valued for their ability to express microbial terroir—much like natural wines or artisanal cheeses—highlighting the living, site-specific biodiversity that shapes flavor as a cultural and ecological expression (23).

From an ethical perspective, fermentation promotes regenerative and sustainable principles by valorizing food waste, extending its shelf life without synthetic additives, and minimizing energy consumption for preservation (24). Furthermore, it enables decentralized, low-tech food production models that foster community resilience and promote gastronomy, especially in rural or resource-limited settings. Thus, fermentation transcends its historical role as a preservation method and is redefined as a central epistemic tool in the fifth gastronomic paradigm, which supports ecological gastronomy, nutritional improvement, and a reinvention of human-microbe collaboration based on ethics, complexity, and coevolution (25).

#### 3.1.3 Artificial intelligence in gastronomy

Artificial Intelligence (AI) is transforming the epistemological landscape of gastronomy, not only through process automation but also by transforming culinary creativity, sensory design, business models, and human-machine interactions (26). In this new paradigm, AI functions not as a passive tool, but as an active agent of culinary innovation. It enables recipe generation, the prediction of novel and culturally hybrid flavor combinations, the optimization of ingredient sourcing, and even the design of entirely new dishes based on nutritional, environmental, and cultural criteria (10, 27, 28).

AI-driven gastronomic designs are based on large-scale chemical, cultural, and sensory datasets, helping chefs design nutritionally balanced, ecologically responsible, and contextually meaningful dishes. This emerging field, called computational gastronomy, enables the recombination of cross-cultural recipes, the prediction of flavor combinations, and the simulation of affective responses to food stimuli (10). These capabilities open the door to a new data-driven aesthetic, where the orchestration of sensory stimuli is guided not only by tradition or intuition, but also by predictive algorithms.

Beyond recipe design, AI-generated visualizations are increasingly being used in the conceptual phase of sustainable gastronomy. As design researchers demonstrate, algorithmically created images allow chefs and designers to explore speculative food futures, reducing material waste and stimulating narrative and aesthetic innovation (29). AI is also advancing the modeling of human taste perception. Algorithms trained with multisensory, chemical, and cultural data can anticipate palatability and suggest novel combinations that challenge traditional gastronomic logic (27). These algorithms do not merely optimize known combinations—they interrogate and surpass gastronomic logic as historically constituted by cultural, regional, and aesthetic conventions. By transcending canonical pairings or ingredient hierarchies, AI invites a rethinking of what constitutes coherence, harmony, or creativity in culinary design. This opens a speculative space where intuition is augmented, but not replaced, by data-driven experimentation and reinforces AI's dual role as a sensory designer and intercultural mediator in the creation of new culinary trends.

Additionally, computer vision technologies have begun to improve the precision of culinary tasks, from the controlled application of edible coatings to the real-time monitoring of fermentation processes, signaling a transition toward precision gastronomy (30). Contemporary food design, as recently reviewed, connects ancient culinary traditions with emerging technological paradigms, such as AI, 3D printing, and biosensing, thus aligning closely with the post-digital integration model proposed in this article (28).

However, the integration of AI also raises complex epistemological and ethical questions. It challenges traditional notions of authorship, originality, and culinary expertise. If algorithms can replicate, or even surpass, human creativity in designing sustainable and emotionally resonant dishes (10), what about the chef's role as author? The transition from artisanal authorship to collaborative creativity between humans and machines requires a redefinition of culinary identity and practice. In this emerging framework, culinary authorship becomes distributed—an assemblage of human intention, algorithmic suggestion, and microbial or material agency. Rather than diminishing the chef's role, this reconfiguration repositions the cook as a curator of multispecies creativity and an orchestrator of entangled intelligences. This post-anthropocentric stance does not erase human creativity but recontextualizes it within ecological, technical, and epistemic networks.

These AI applications also support the broader transformation of artificial foods through 3D printing and the intelligent formulation of new proteins. This convergence of AI and food biotechnology embodies the multispecies and epistemologically transformative goals of the fifth gastronomic paradigm (31).

The creation of novel foods itself—which intersects gastronomy, biotechnology, and interaction design—emerges as a transdisciplinary language where AI facilitates the articulation of aesthetic, ecological, and ethical meanings through edible agents. In this context, gastronomy becomes a means of knowledge production, and AI becomes a co-author of posthuman culinary narratives. AI-based culinary platforms, such as NotCo or Brightseed, exemplify how algorithmic systems can recombine molecular, nutritional, and sensory data to generate novel foods aligned with more regenerative and healthy values (32).

Recent contributions underscore that AI not only optimizes ingredients, flavor, and texture but also catalyzes a broader transition toward equitable and sustainable food systems. According to Kuhl, artificial intelligence accelerates food innovation by correlating chemical composition with sensory perception, predicting functional properties, and formulating new gastronomic constructs based on computational cues (32). These capabilities could not only support chefs and researchers but would transform gastronomy into a distributed, cognitive system enhanced by layers of big data previously inaccessible or invisible to the cook.

Taken together, these innovations reflect a radical restructuring of culinary knowledge. The integration of AI into gastronomy marks a shift from human-centered cuisines to an integrative epistemology in which human, artificial, and microbial intelligence converge to shape the future of food. Within the fifth gastronomic paradigm, AI emerges not only as a tool but as a generator of culinary creativity, enabling a new form of gastronomy that is simultaneously sensorial, scientific, and systemic. In this framework, AI no longer operates in the background of culinary systems: it emerges as a generative force that redefines not only what we eat, but how knowledge, culture, and ethics are encoded into the very act of cooking.

### 3.2 Regeneration as culinary value

Within the fifth gastronomic paradigm, regeneration becomes a central epistemic and operational principle, transcending trend cycles to redefine the ontological and ethical foundations of the gastronomy of the future. Regenerative gastronomy goes beyond the notion of sustainability by seeking not only to reduce environmental impact, but also to actively restore ecosystems, enhance biodiversity, and repair the social fabric linked to food systems. It is based on principles that align food production, processing, and consumption with the rhythms and resilience of living systems, reconfiguring gastronomy as a form of ecological intelligence that operates through taste, technique, and territorial integration. Central to this perspective is Loring's theoretical framework, based on the “conservation of change”—a concept derived from thermodynamics—which posits that food systems can only be regenerative when they incorporate variability, diversity, and feedback loops, rather than suppressing them through industrial standardization (33). This understanding aligns with practices already visible in the culinary field: chefs who embrace regenerative principles prioritize seasonality, polyculture, heirloom varieties, and partnerships with smallholder producers who practice ecological management. Ingredients are sourced from regenerative agriculture, agroforestry, and permaculture initiatives that restore soil health, sequester carbon, and sustain resilient local economies. In this framework, gastronomy becomes a platform for ecological repair—not merely reflecting but enacting regenerative values through sourcing, technique, and multispecies collaboration.

Empirical data from southern and eastern Africa reinforce the viability of this model. Moodley et al. (34) show that regenerative agriculture in South Africa offers climate-smart and soil-restorative strategies essential for long-term food security, especially in regions at risk of desertification and land degradation. Similarly, Ntawuhiganayo et al. (35) demonstrate that household food security improves significantly when regenerative practices such as crop rotation, agroforestry, and circular nutrient flows are adopted in combination. Moreover, gastronomic regenerative approaches have been associated with improved nutritional outcomes and wellbeing, demonstrating that these principles can inform not only agricultural practices but also culinary creativity and product development (36).

Beyond food sourcing and production, regenerative gastronomy reimagines the relationship between plate and territory, fostering a culinary narrative that not only reflects but also intervenes in the socio-ecological dynamics of a restaurant. Menus are conceived as vehicles for food justice, supporting marginalized communities and advocating for equitable access to food. Initiatives such as the FixOurFood project exemplify how regenerative transformation can operate at multiple scales, from agricultural practices to institutional and policy frameworks, turning regeneration not just into a technique but into a systemic paradigm (37). Chefs such as Dan Barber (USA) and Rodrigo de la Calle (Spain) (see case studies below) exemplify this regenerative shift, developing menus that prioritize soil health, agricultural polyculture, and biodiversity as gastronomic values. By centering regeneration as a culinary value, this paradigm reframes the act of cooking as a contribution to planetary healing, reframing gastronomy as a transdisciplinary praxis that mediates between ecological repair, cultural identity, and systemic food transformation. However, the implementation of regenerative gastronomy is not without limitations and contradictions. Despite its ecological aspirations, many regenerative practices still rely on high-end logistics, niche markets, and the symbolic capital of renowned chefs, limiting their scalability and accessibility. Furthermore, the commodification of regeneration—through marketing narratives or superficial claims of sustainability—risks diluting its transformative potential. Authentic regenerative gastronomy requires not only technical adjustments or aesthetic reformulations, but also demands structural reform across supply chains, food education, and governance mechanisms. Therefore, its consolidation as a paradigm depends on bridging the gap between visionary prototypes and systemic integration. In this sense, regenerative gastronomy exemplifies the epistemological shift of the fifth paradigm: from extractive models of culinary excellence to participatory systems of ecological intelligence.

### 3.3 Gastronomy as reduction: from excess to essence

This section explores how the fifth paradigm redefines the materiality of gastronomy. Rather than embracing theatrical excess or elaboration, emerging culinary practices increasingly privilege reduction, subtlety, and essence. Food is no longer limited to the plate as a static object but becomes part of an immersive and interactive system that engages the senses, cognition, and imagination. Technologies such as augmented reality, virtual reality, haptic interfaces, and soundscapes allow chefs to manipulate perception, texture, and emotions, transforming the gastronomic experience into a narrative and experiential act. In this emerging landscape, chefs become designers of immersive experiences that transcend the restaurant itself, acting as cognitive engineers and facilitators of multisensory episodes. Perullo and others conceptualize this shift as a movement from optical to haptic taste, privileging the relational, immersive, and ecological dimensions of aroma, touch, and flavor over visual consumption and commodification (3, 38). This philosophical reorientation challenges contemporary gastronomic criticism as too superficial and demands a deeper aesthetic and cognitive engagement with the cook from the diner. The phenomenon of digital cuisine exemplifies this evolution: chefs now use virtual storytelling, simulation environments, and sensory amplification to construct gastronomic meaning without relying on the physical presence of the food itself (39). Historical precedents such as elBulli foreshadowed this trend through some of Ferran Adrià's experiments and, crucially, through the then-called techno-emotional cuisine not only of Adrià but even more so of Heston Blumenthal, where food unfolded with numerous symbolic gestures and affective provocations (1, 40–42). Furthermore, social media platforms have become central to this process of dematerialization. Empirical research in Serbia indicated that digital representations of food significantly influence restaurant choice and perceived quality, sometimes more than the physical experience itself (43). Branding studies confirm that networks such as Instagram and TikTok function as dematerialized arenas, where gastronomic value is produced through visual storytelling, abstraction, and the symbolic capital of the act, rather than through sensorial authenticity (44).

Beyond aesthetic and technological novelty, gastronomic dematerialization carries significant ethical and ecological implications. By dissociating sensorial satisfaction from material abundance, it allows for a redefinition of luxury in non-extractive terms, prioritizing affective design, narrative depth, and multisensory immersion over rare or resource-intensive ingredients. This change helps reduce food waste, lower the carbon footprint of global sourcing, and rethink luxury with ecological care. In this sense, dematerialized gastronomy not only challenges traditional culinary hierarchies but also aligns itself with regenerative ethics by proposing new models of experiential abundance at a lower environmental cost. Nevertheless, one should not overlook the paradox that immersive fine dining experiences often require long-distance travel by affluent diners, significantly increasing the carbon footprint of dematerialized gastronomy.

Ultimately, gastronomic dematerialization opens new avenues for ethical and ecological innovation by dissociating sensorial satisfaction from material excess, inviting reflection on the nature of pleasure, identity, and the future of food (3). Far from representing a frivolous departure from material food, gastronomic dematerialization—when aligned with ethical intent and environmental reflection—offers a path to reimagine pleasure, aesthetics, and meaning beyond intensive resource consumption, positioning itself as an important strategic and epistemological tool within the fifth gastronomic paradigm.

Far from being a frivolous detachment from material food, gastronomic dematerialization—when guided by ethical and ecological reflection—offers a path to reimagine pleasure and meaning through low-impact experiences, positioning itself as a strategic and epistemological lever in designing future food cultures.

### 3.4 Crisis-responsive cuisine

Crisis-Regulated Cuisine has emerged in direct response to the escalating environmental, social, and geopolitical disruptions of the 21st century. The COVID-19 pandemic highlighted weaknesses in global food systems and exposed the fragility of haute cuisine. Harba et al. (45) document how these crises caused not only an economic shock—with revenue drops of over 85% in high-end restaurants—but also a profound shift in consumer behavior and emotional expectations, transforming perceptions of security, trust, and hospitality itself.

In this context, haute cuisine began to reorient itself around the principles of adaptability, resource efficiency, and resilience. Crisis management expanded beyond short-term continuity to incorporate ethical sourcing, local supply chains, and digital transformation strategies (46). This reorientation positions gastronomy less as a luxury pursuit and more as a systemic framework of public value attuned to planetary volatility.

Crisis-driven gastronomy increasingly embraces resilient and unconventional ingredients—such as algae, insects, mycoproteins, and upcycled waste—that were once dismissed as marginal. These are now reframed as symbols of ecological adaptation and gastronomic innovation, integrating perspectives from both ancestral strategies and cutting-edge research, incorporating food systems analogous to spatial ones or molecular recycling (47). Scarcity is no longer seen as a limitation, but rather a catalyst for creativity, sustainability, and a new ethic of sufficiency.

Recent contributions further emphasize that food crises are not only logistical or nutritional, but also deeply symbolic and emotional. As Gracia-Arnaiz (48) points out, the pandemic catalyzed a transformation in Spanish consumption habits, redirecting them from hedonistic and identity-based consumption toward more thoughtful, healthy, and ecologically conscious eating practices. This reorientation underscores gastronomy's emerging role as a vehicle for cultural resilience and ethical recalibration.

A crucial aspect of this transition is the growing volume of strategic culinary planning geared toward crisis adaptation. Internal reports highlight the importance of proactive protocols, stakeholder communication, and flexible operating models to maintain functionality during supply chain disruptions, pandemics, or geopolitical instability (49). Technological responses include algae-based proteins—ecologically efficient and compatible with circular models—that offer scalable nutritional solutions (50) (see below). Likewise, the development of 3D and 4D food printing technologies allows for personalized nutrition, adaptive textures, and storage resilience, particularly valuable in emergency or resource-scarce contexts (51, 52).

Furthermore, alternative proteins derived from mushrooms, legumes, and cultured meat are increasingly explored as models for post-scarcity food systems. Though still in early stages of adoption, these technologies aim to reconfigure gastronomy around the anticipation of crisis as a structural condition of the Anthropocene, rather than an episodic anomaly (53).

In sum, crisis-responsive cuisine exemplifies the fifth gastronomic paradigm's commitment to systemic transformation. By embracing sufficiency, humility, and resilience—not as limitations, but as affirmative values—it redefines gastronomy as a domain of anticipatory ethics and planetary design. It invites chefs to act not merely as artisans of taste, but as stewards of ecological foresight and agents of cultural reinvention.

#### 3.4.1 Algae and plankton-based gastronomy

Algae-based gastronomy constitutes a key vector of the fifth gastronomic paradigm, blending high nutritional value with exceptional ecological sustainability. Macroalgae and microalgae provide complete proteins, essential fatty acids, and bioactive micronutrients, making them ideal components of a forward-looking, marine plant-based cuisine. Their minimal environmental footprint and compatibility with circular economy principles position them as agents of systemic food transformation. Nonetheless, their widespread adoption remains limited by cultural preferences and regulatory barriers, especially in Western markets. Initiatives such as EU4Algae aim to overcome these challenges by promoting innovation, regulatory harmonization, and expanded culinary applications beyond traditional uses (50, 54).

Recent breakthroughs in algal fermentation are expanding their gastronomic potential. Fermentation improves digestibility and enhances the bioavailability of polyphenols, peptides, and vitamins, while modulating marine flavors and yielding more accessible sensory profiles. Reboleira et al. (55) demonstrate how lactic acid bacteria, yeasts, or mixed cultures can produce creamy textures with distinctive iodine and marine aromas balanced by mild acidity, rendering them ideal for haute cuisine. These microbial processes also facilitate the incorporation of algae into foundational food categories, including plant-based or cultured meats, dairy analogs (fermented or non-fermented), and an expanding range of gourmet condiments (55).

Beyond functionality, algal fermentation contributes to microbial biodiversity and the valorisation of underutilized marine biomass. Chefs and scientists are increasingly exploring “fermentation design” as a vehicle for integrating oceanic ecological narratives into gastronomy—treating fermentation not merely as a technique, but as a medium of regenerative storytelling (55).

The gastronomic relevance of seaweed also encompasses cultural and territorial dimensions. Merkel et al. (56) highlight how artisanal seaweed harvesting sustains coastal economies, preserves traditional ecological knowledge, and fosters innovation in marginal marine regions. Their work advocates for value chains that respect ecological rhythms and community sovereignty, redefining seaweed as both an ingredient and a socio-ecological resource (56). Technological advances such as encapsulation, extrusion, and enzymatic hydrolysis have further expanded seaweed's culinary applications, allowing its integration into breads, pastas, foams, and desserts. These biotechnological tools enhance both nutritional value and creative potential. The umami intensity, tactile diversity, and vibrant colors of algae offer chefs a refined sensory palette that evokes an aesthetic ethically linked to marine ecosystems and ecological purity.

As highlighted in recent literature, microalgae—including Spirulina, Chlorella, and Nannochloropsis—are increasingly used in haute cuisine not as hidden supplements, but as culinary protagonists. Their pigments serve as natural colorants, while their distinctive flavors provide depth and originality. This shift reflects an evolution in perception: microalgae are no longer emergency foods, but symbolic agents of post-anthropocentric and regenerative gastronomy (56, 57).

Aponiente, the three-Michelin-starred restaurant led by Ángel León in Cádiz, Spain, exemplifies the gastronomic potential of marine plankton as both an ingredient and narrative tool. Through the biotechnological stabilization of phytoplankton—rich in omega-3s, minerals, and pigments—Aponiente has incorporated this microscopic biomass into sauces, foams, and broths, offering a direct sensory expression of marine ecology. This approach resonates with the post-anthropocentric and regenerative values of the sixth gastronomic paradigm, repositioning plankton from a technical additive to a culinary medium that conveys scientific insight, ecological responsibility, and sensory innovation (58).

#### 3.4.2 Insect cuisine

Insect cuisine has evolved from a marginal cultural curiosity to a critical component of crisis-responsive gastronomy within the emerging fifth paradigm. As global food systems face escalating pressures from environmental degradation, population growth, and nutritional inequality, edible insects present a scalable, resilient, and ecologically sound alternative protein source. Halloran et al. emphasize that the incorporation of insects into gastronomy not only advances sustainability goals but also expands the culinary imagination by enriching the sensory and symbolic dimensions of food cultures (59).

The nutritional profile of insect-based products is particularly compelling: powders derived from crickets or mealworms deliver high-quality proteins alongside essential micronutrients such as iron, calcium, and zinc, while requiring significantly less land, water, and feed than conventional livestock systems (60). From an environmental perspective, their low greenhouse gas emissions and circular production potential position them as a cornerstone of low-impact protein strategies.

However, the so-called “disgust barrier” remains a critical challenge to widespread adoption, particularly in Western contexts. Nevertheless, consumer research shows that this barrier can be mitigated through increased exposure, trusted branding, and thoughtful culinary recontextualization. Processed formats—such as insect-based pastas, protein bars, or sauces—tend to elicit higher consumer acceptance than whole-insect presentations, especially when framed by narratives of health, sustainability, or innovation (61).

Notably, recent studies suggest that culinary students—future chefs and gastronomic influencers—demonstrate increased openness to edible insects when these ingredients are introduced in professional training settings that emphasize sustainability and creative innovation (62, 63). This educational exposure fosters a generational shift in food aesthetics and ethics, positioning emerging culinary professionals as key agents in normalizing insect cuisine within both haute cuisine and everyday food cultures.

As climate adaptation becomes a defining feature of future food systems, insect gastronomy exemplifies how culinary creativity can engage with protein scarcity, ecological urgency, and cultural transformation. By reframing insects not as fallback options but as versatile, ethical, and culturally expressive ingredients, this subfield embodies the anticipatory, regenerative, and pluralistic ethos of the fifth gastronomic paradigm.

### 3.5 Collective intelligence and hybrid knowledge systems

In the emerging fifth gastronomic paradigm, the production of culinary knowledge is undergoing a profound transformation. No longer the exclusive domain of individual chefs, elite institutions, or Western epistemologies, knowledge now emerges from complex and largely delocalized networks of interdisciplinary collaboration. This reflects a decisive shift toward collective intelligence, through which chefs, scientists, designers, foragers, indigenous communities, and technologists co-create evolving ecosystems of knowledge and practice. In this framework, the chef ceases to be a solitary author and becomes a cultural mediator: a node within a collaborative system of meaning-making that integrates diverse forms of knowledge and worldviews. This transformation is visible in experimental restaurants like Mugaritz, foods design hubs like Space10, and numerous interdisciplinary food labs (see Case Studies below), where gastronomic innovation merges microbial science, artistic discourse, artificial intelligence, ancestral techniques, and immersive sensory storytelling. These initiatives exemplify the consolidation of hybrid knowledge systems: structures that transcend the duality between traditional and scientific knowledge, or between empirical practice and technological abstraction. In this context, regenerative agriculture practices, fermentation, biodiversity preservation, and biotechnological development are not treated as isolated domains, but as intertwined layers of a shared epistemic project.

This shift is not merely rhetorical, but structural. Culinary R&D increasingly operates in teams that combine expertise in microbiology, sustainability science, sensory analysis, food design, environmental humanities, and cultural anthropology. As Mestre et al have observed, these teams exemplify new collaborative logics that require the negotiation of disciplinary boundaries and epistemological frameworks (64). Knowledge hybridization thus becomes not only a methodological strategy but a structural condition of contemporary gastronomy: a reflection of the complexity of the food systems with which it interacts and which it seeks to transform.

The logic of integration extends even further. In recent years, authors such as Galarraga and Albeniz have emphasized that current gastronomic creativity emerges from “dynamic boundary work” between heterogeneous epistemic communities, which entails nonlinear processes of negotiation and translation between practices as diverse as molecular biology, digital fabrication, anthropological fieldwork, and art installation (65). These interactions are not only productive in terms of outcomes but also constitute a new epistemological model that reframes gastronomy as a knowledge system that is situated, reflexive, and transdisciplinary by design.

Crucially, collective intelligence in gastronomy increasingly transcends human agency. As Díaz and Albeniz argue, microbial agents, fermentation cultures, technological infrastructures, and generative AI are increasingly central to gastronomic ecosystems (66). In this sense, the use of generative AI in the writing of this article aligns with the paradigm's epistemological logic. Rather than replacing authorial agency, it reflects a hybrid and dialogical form of concept development—one that echoes the distributed, cross-intelligent processes seen in contemporary culinary laboratories. Its inclusion is therefore both methodological and philosophical: a lived expression of the very paradigm it helps to describe. These assemblages call for a reconceptualization of culinary practice as a multi-species post-anthropocentric system of knowledge production. Within such frameworks, the boundary between nature and culture, or between craft and computation, becomes increasingly porous.

The implications of this shift are also philosophical. As Albeniz note in their call for a “gastrology” rather than a mere gastronomy, contemporary food innovation demands a complexity-oriented approach, embracing open systems thinking and rejecting linear or reductionist frameworks (67). Gastrology is thus positioned not as a new discipline but as an epistemic disposition: a way of understanding food through its entanglement with ecology, politics, ethics, and semiotics. This resonates with the fifth paradigm's emphasis on regeneration, cooperation, and holistic integration.

Additionally, some authors highlight the strategic value of multidisciplinary creative teams in gastronomy laboratories, where chefs collaborate with artists, designers, and scientists to develop experiential food services. These environments act as incubators for novel sensory and symbolic repertoires, enabling the co-creation of gastronomic formats that respond to complex social demands (46, 66, 68). The design of experiences—rather than mere dishes—becomes a central vector innovation pushing gastronomy into the realm of speculative design and cultural prototyping and reinforcing the idea that gastronomy is now a cultural technology operating across sensorial, emotional, and cognitive domains.

Such transformations are not frictionless. The interphase between scientific rigor and creative intuition requires active negotiation of power asymmetries and epistemic hierarchies. As Guixer shows, successful collaboration across culinary and scientific domains depends on dialogic attitudes, epistemological humility, and shared protocols of experimentation (69). This suggests that future training models in gastronomy should incorporate not only technical expertise but also meta-disciplinary literacy—the ability to move fluently between languages, methodologies, and ontologies.

Holistic cuisine, as articulated by recent scholarship (70), encapsulates the philosophical and operational core of the fifth gastronomic paradigm. It redefines cooking as an expanded field of ethical, aesthetic, ecological, and relational inquiry—where taste is only one of many channels through which meaning is conveyed. This approach embraces a wide spectrum of knowledge and affects, weaving together artistic performance, cultural memory, scientific research, sensory immersion, sustainability goals, and social critique. Rather than treating food as a commodity or a spectacle, holistic cuisine constructs it as a medium for multispecies coexistence, experiential depth, and planetary care. Within this framework, the act of dining becomes a transformative ritual that engages mind, body, and environment, reflecting a systemic ethics of nourishment.

Ultimately, this epistemic reconfiguration aligns with Kuhn's understanding of paradigmatic science as a community-driven endeavor, one where consensus is constructed through evolving practices and shared commitments. This epistemic transformation in gastronomy increasingly incorporates aesthetic, performative, and ecological dimensions. As Abrams proposes in his concept of “ecological dramaturgy,” the plate itself may function as a landscape device—staging environmental relationships, cultural memory, and affective meanings within the act of dining (71). This theatrical framing aligns closely with Mugaritz's ontological provocations, where dishes operate as philosophical artifacts; with Atelier Crenn's poetic activism, where food becomes a canvas for feminist and ecological storytelling; and with Alchemist's immersive “impressions,” which explicitly stage global crises such as climate change, social injustice, or animal exploitation. These dining experiences resist the commodification of food by embedding it in a narrative logic that links eaters, ingredients, and landscapes through participatory meaning-making. In this way, gastronomy evolves into a multisensory interface for exploring the entanglement of human and non-human agencies, fully in tune with the fifth paradigm's ethic of interconnectedness and symbolic reparation.

The fifth gastronomic paradigm, viewed through this lens, reframes culinary innovation as a post-disciplinary, regenerative praxis—one that dissolves inherited boundaries between disciplines, cultural systems, and biological categories to cultivate a more reflective, inclusive, and sustainable relationship with food. As summarized in Table 2, the fifth paradigm is defined by five interdependent dimensions.

**Table 2:** Core dimensions of the fifth gastronomic paradigm.

**Dimension**	**Description**
Regenerative Ethics	Centering food systems around ecological repair and systemic care
Biotechnological Integration	Use of AI, fermentation, and precision food design tools
Hybrid Knowledge Systems	Collaboration across science, design, indigenous knowledge, and gastronomy
Multispecies Agency	Inclusion of microbes, plants, AI, and non-human actors in creativity
Anticipatory Imagination	Culinary futures prototyped through scenario design and narrative gastronomy

Each dimension expresses a systemic shift in the epistemology, ethics, and aesthetics of contemporary gastronomy, with emphasis on regeneration, collaboration, and multispecies agency.

### 3.6 Case studies

A constellation of pioneering gastronomic projects around the world exemplifies the principles of collective intelligence, hybrid knowledge systems, and postdisciplinary innovation at the heart of the emerging fifth gastronomic paradigm. These initiatives transcend gastronomy's legacy as the exclusive domain of individual genius, repositioning it as a collaborative and regenerative system where culinary creation emerges from the intersection of science, ecology, ethics, and cultural heritage.

In Spain, Mugaritz (Andoni Luis Aduriz) stands as a paradigmatic culinary laboratory. For more than two decades, it has operated as a space for epistemic experimentation, where gastronomy is redefined through microbial research, posthumanist aesthetics, philosophical dialogue, and sensorial provocation. Mugaritz explores food not only as sustenance or pleasure, but as a vehicle for cognitive and emotional disruption, reframing gastronomy as a tool for ontological inquiry and systemic reflection (72). Beyond its technical innovations, Mugaritz consciously constructs an identity that blurs the boundaries between haute cuisine, popular culture, and artistic discourse, challenging traditional notions of authorship and prestige (73). Its emphasis on process over product and uncertainty about mastery situates the restaurant within a new epistemological regime, where the chef ceases to be a transmitter of codified knowledge and instead facilitates open-ended, collaborative epistemic exploration. Mugaritz exemplifies the integration of epistemological realignment, multispecies agency, and anticipatory imagination. Its open-ended research model and philosophical framing situate it at the core of the paradigm's post-disciplinary logic.

Aponiente (Ángel León), also in Spain, exemplifies the regenerative spirit of the fifth paradigm through its radical focus on marine ecosystems. Integrating oceanographic research, traditional fishing knowledge, and biotechnological innovation, Aponiente transforms forgotten marine species and algae into haute cuisine. His practice includes the creative use of microalgae such as Tetraselmis and Spirulina, harnessed not only for their sustainability and nutritional value, but also for their expressive visual and sensorial properties (57). Beyond gastronomy, Aponiente acts as a platform for ecological activism and scientific outreach, in line with the CSC (Cuisine-Science-Communication) model, where the kitchen becomes a space for scientific narrative and environmental awareness (58). At Aponiente, cuisine is not only food, but a platform for marine regeneration, cognitive renewal, and ecological storytelling rooted in post-anthropocentric ethics (74). Aponiente reflects regenerative ethics, multispecies agency, and hybrid knowledge systems through its focus on marine ecology, biotechnological experimentation, and oceanic restoration.

Likewise, Azurmendi (Eneko Atxa) embodies a systemic model of culinary sustainability. Located in a bioclimatic building, the restaurant integrates renewable energy, circular waste management, local biodiversity restoration, and social innovation programs. Their collaborative projects with environmental scientists, farmers, and educators position Azurmendi as an integrated culinary ecosystem in its territory that reflects the fifth paradigm's ethic of care and participation (75). Notably, a recent research project co-authored by Atxa explores the recycling of food waste, such as leftover bread, to make sustainable non-alcoholic carbonated beverages through microbial fermentation, exemplifying Azurmendi's commitment to circular gastronomy and low-impact biotechnologies (76). This initiative not only minimizes food waste but also connects scientific research with culinary creativity, reaffirming the restaurant's role as a living laboratory where gastronomy contributes to ecosystem regeneration and collaborative innovation. Azurmendi exemplifies the intersection of regenerative ethics and hybrid systems, embodying ecological care through infrastructural design, circular innovation, and biocultural partnerships.

Noor (Paco Morales) exemplifies a nuanced case of decolonial gastronomy. The restaurant reconstructs Andalusi culinary heritage through rigorous historical research, collaboration with Arab and Mediterranean scholars, and symbolic storytelling. By recovering lost ingredients, techniques, and epistemologies, Noor challenges hegemonic narratives and offers a form of cultural restoration and symbolic sovereignty. While it does not foreground biotechnology or ecological regeneration, its focus on gastronomic historicity aligns with the fifth paradigm's emphasis on cultural and epistemic regeneration. Morales' approach, described as “gastroarchaeology,” involves meticulous archival research and collaboration with food historians, ensuring that each dish authentically reflects the culinary practices of Al-Andalus (77). This commitment to historical accuracy and cultural dialogue positions Noor as a culinary ecosystem embedded in its territory and reflective of the fifth paradigm's ethic of situated care and cultural agency. Noor highlights the dimension of epistemological realignment and situated cultural agency, emphasizing historical recovery and symbolic sovereignty as tools of gastronomic regeneration.

Across the rest of Europe, Alchemist (Rasmus Munk, Denmark) stands out as one of the most radical embodiments of the fifth gastronomic paradigm. Its concept of Holistic Cuisine transcends the culinary act by integrating performative art, scientific research, social activism, and immersive technology. Each of its “impressions”—highly conceptual dishes—addresses global issues such as climate change, food injustice, or animal suffering, transforming dining into a vehicle for ethical and sensory disruption. Through its Spora Lab, Alchemist collaborates with academic institutions in experimental projects such as stratospheric cooking and the development of shape-changing edible materials, fostering new sensory interfaces between food and diners (78). Studies conducted at Alchemist further demonstrate how emotional context modulates taste perception, highlighting the restaurant's multisensory, affective impact demonstrating how affective multisensoriality becomes a medium for socio-political resonance (79). These immersive experiences redefine gastronomic intermediality as a form of critical communication (78). Simultaneously, initiatives like JunkFood, offering nutritious meals to marginalized populations, exemplify Alchemist's tangible commitment to social justice.

Alchemist activates anticipatory imagination, multisensory design, and regenerative ethics through immersive gastronomy, critical aesthetics, and technological speculation. In contrast, Ultraviolet (Shanghai), while pioneering in multisensory dining, centers on secrecy and performative control that renders gastronomy subordinate to theatrical illusion (80). Ultraviolet's emphasis on theatrical illusion, absent any ecological or social commitment, underscores the limitations of experiential gastronomy when severed from the regenerative ethics central to the fifth paradigm.

Noma (René Redzepi, Denmark) stands out as a pivotal case in the paradigm shift toward post-disciplinary and regenerative gastronomy. In its later stages, the restaurant moved away from the conventional service model to become a culinary laboratory dedicated to sensory research, Nordic harvesting, microbial fermentation, and interdisciplinary collaboration with biologists, anthropologists, designers, and perfumers (81). Its exploration of microbial symbiosis, underutilized ingredients, plant ecology, and climate narrative transcended the stylistic and sensory frontiers of the fourth paradigm, signaling a shift toward ecological intelligence and epistemic integration, positioning Noma as an experimental node for the new culinary epistemology. Although it closed its doors as a traditional restaurant, its institutional legacy lives on through the Noma Projects, which continue to develop food research, education, and scientific communication through gastronomy (82). As Mejía and Wilson (83) have shown, this transformation also emerges from a critical re-evaluation of the social unsustainability of haute cuisine, especially in terms of labor, exclusivity, and cultural hegemony, affirming the fifth paradigm's demand for an ethical, systemic, and epistemological redefinition of what haute cuisine can be.

Finally, in Europe, Space10 (Denmark), an experimental laboratory founded by IKEA and active between 2015 and 2023, functioned as a speculative engine for post-disciplinary food futures. Through projects focused on 3D-printed foods, algae-based proteins, closed-loop food systems, and food democracy, it functioned as an innovative hub of culinary imagination. Its cookbook, Future Food Today (84), presents not only recipes but also scenarios that explore how design, technology, and local ecosystems can shape the meals of the future. The lab's open source and participatory ethos fostered citizen engagement and democratized food innovation, addressing planetary challenges such as climate change, over-urbanization, and resource scarcity. Although no longer operational, Space10 remains relevant as a reference model for interdisciplinary research and food design that transcends current haute cuisine, offering a blueprint for the speculative, collaborative, and transdisciplinary ethos that defines the fifth gastronomic paradigm. Space10 exemplified anticipatory imagination, post-disciplinary integration, and democratization of innovation, aligning design, food systems, and open-source futures.

In the Americas, Atelier Crenn (Dominique Crenn, USA) offers a poetic and ecologically grounded reimagination of haute cuisine, deeply aligned with the fifth gastronomic paradigm. The restaurant's menus are conceived as narrative landscapes—reflections of memory, place, and interdependence—where ingredients are not mere culinary elements but participants in a larger ecological dramaturgy (71). Each dish functions as a multisensory composition that invokes origin, sustainability, and affective resonance. Crenn's cuisine emphasizes an ethos shaped by regenerative agriculture and biographical storytelling, free of animal flesh and centered on biodiversity. This aligns with Abrams' notion of the “plate as landscape device,” in which the gastronomic experience becomes a performative medium for environmental and cultural expression (71). In this way, Crenn's work not only nourishes but also transforms the act of eating into a theatrical, ethical, and planetary gesture (85). Atelier Crenn integrates regenerative ethics, multispecies aesthetics, and biographical ecology, offering a poetic expression of post-anthropocentric gastronomy.

Also in the United States, Owamni (Sean Sherman) manifests the fifth paradigm through a radical decolonial approach. Recovering precolonial ingredients and Indigenous knowledge, the project affirms culinary sovereignty, environmental reparation, and cultural resurgence. Gastronomy here is a epistemic tool of resistance and healing, integrating food harvesting, seed preservation, and Indigenous science, even if expressed through a modest serving format (86). Owamni illustrates epistemological realignment and regenerative ethics through decolonial gastronomy, Indigenous science, and culinary sovereignty.

In South America, Rodolfo Guzmán has developed a model of endemic cuisine at Boragó that goes far beyond aesthetic narrative. In collaboration with indigenous communities, farmers and local gatherers, he has systematically documented hundreds of native ingredients, creating a cuisine based on extreme seasonality and territorial adaptation (87). Notably, Guzmán's collaboration with food scientist José Miguel Aguilera connects Boragó to a deeper scientific lineage. Aguilera's foundational articles on the birth of gastronomic engineering (88, 89) provided a framework for integrating physicochemical analysis, sensory design, and culinary creativity—anticipating the very hybrid knowledge systems that define the fifth gastronomic paradigm. Boragó brings together hybrid knowledge systems, regenerative ethics, and epistemological realignment, grounded in radical seasonality, scientific partnership, and biocultural research.

From Asia, Native (Vijay Mudaliar, Singapore), in his modest bar, reframes the city as a foraging ecosystem. His menu features urban botanicals, fermented insects, and waste valorization strategies that reimagine culinary sourcing beyond industrial models. Scientific literature has highlighted the potential for gastronomic innovation of edible insects when integrated through fermentation and sensory refinement (90), as well as the cultural and ecological role of urban foraging in tropical contexts (91). Native integrates circular gastronomy with sensory experimentation and local ethnobotany, articulating a distinctly urban and tropical inflection of the fifth gastronomic paradigm. Native synthesizes urban foraging, circular gastronomy, and multispecies engagement, embodying an ecologically adapted and resource-aware expression of the paradigm.

From Oceania, Attica (Ben Shewry, Australia) applies similar principles in a haute cuisine format, cultivating local herbs and utilizing sustainable seafood and insects. Attica similarly synthesizes circular gastronomy with ecological precision and multispecies aesthetics, applying these principles in a high-end culinary context rooted in coastal Australia. Attica reflects regenerative ethics and multispecies aesthetics through local sourcing, insect use, and coastal biodiversity as material for ecological narrative.

Finally, in Africa, Midunu (Selassie Atadika, Ghana) represents a prominent example of African gastronomic epistemologies within a regenerative logic. Her cuisine is based on traditional West African fermentation, millet-based systems, and climate-adapted ingredients. By connecting culinary anthropology, sustainability, and social entrepreneurship—particularly involving women farmers—Midunu contributes to a branch of the fifth paradigm, one that is underrepresented globally but epistemologically rich. As highlighted in a Harvard Business School case study, Atadika's innovation lies not only in the use of ancestral ingredients and narrative scenes, but also in the creation of premium products such as artisanal chocolates infused with local herbs and spices (92). Midunu embodies regenerative ethics, epistemological realignment, and hybrid knowledge systems, combining ancestral fermentation, climate-adaptive crops, and social entrepreneurship.

Although dispersed across continents and formats, these case studies converge in their commitment to plural epistemologies, ecological regeneration, ethical coherence, and systemic transformation. Their practices confirm that gastronomy, reframed as a distributed knowledge system, can act not merely as a site of creative expression, but as an infrastructure for cultural reparation, environmental stewardship, public health innovation, and biotechnological speculation—foundational dimensions of the fifth gastronomic paradigm. Table 3 presents selected examples that exemplify this paradigm across diverse socio-ecological contexts.

**Table 3:** Case studies and paradigmatic features.

**Project**	**Country**	**paradigm features highlighted**
Mugaritz	Spain	Microbial co-creation, philosophical gastronomy
Aponiente	Spain	Marine regeneration, biotechnology, oceanic storytelling
Azurmendi	Spain	Circular sustainability, socio-ecological design
Atelier Crenn	USA	Feminist ethics, narrative aesthetics, regenerative agriculture
Owamni	USA	Decolonial food systems, indigenous knowledge and sovereignty
Alchemist	Denmark	Holistic cuisine, multisensory speculation, social justice
Boragó	Chile	Endemic gastronomy, ethnobotany, foraging and scientific collaboration
Midunu	Ghana	African epistemologies, agroecological chocolate, climate adaptation
Native	Singapore	Urban foraging, circular gastronomy, insect fermentation
Attica	Australia	Sustainable seafood, terroir-based creativity, digital fermentation

## 4 Discussion: gastronomy as transformative praxis

### 4.1 Persistent tensions and the incomplete consolidation of the paradigm

The emergence of the fifth gastronomic paradigm—grounded in regenerative ethics, post-digital aesthetics, and biotechnological innovation—has not yet crystallized into a unified or broadly institutionalized transformation. While academic literature offers a theoretical framework for understanding this shift, professional discourse and media interviews provide complementary insight into how chefs, critics, and institutions perceive, negotiate, and narrate the evolving paradigm. Though not peer-reviewed, such commentaries surface lived tensions, aspirations, and ambivalences that shape the culinary field from within. Despite the widespread rhetorical commitment to sustainability, few chefs consistently translate ecological or public health principles into practice. As one study observes, “Chefs have the potential to influence dietary quality and the sustainability of food systems… yet we know little about chefs' pro-environmental behaviors and attitudes toward reducing food waste and GHG emissions” (93). This inertia reflects the enduring influence of the previous paradigm of Culinary Abstraction, which prioritized spectacle, individual authorship, and sensory provocation. Rather than a smooth transition, the current moment illustrates a paradigmatic disjunction marked by contradictions between ethical aspiration and entrenched professional habitus.

A particularly illustrative tension lies in the reception of cultured meat. This technology promises to dissociate animal suffering from protein production, aligning itself with post-anthropocentric ethics. However, unresolved concerns about sensory authenticity, nutritional value, long-term safety, and techno-solutionism persist (15, 17, 20). Beyond technical feasibility, it has been emphasized that public perceptions of lab-grown meat are deeply intertwined with symbolic and cultural meanings associated with ‘real meat,' which cannot be easily replicated by cellular technologies (15). This reflects a broader truth: technoscientific innovation, in isolation, cannot overcome the deep symbolic architecture of food culture.

Meanwhile, others point out that without integrating these innovations into a broader ethical and social dialogue, they risk reproducing the same structural flaws as industrial meat systems (17). These doubts reveal a deeper problem: technological progress alone cannot substitute for the cultural, symbolic, and affective dimensions that have historically shaped food systems.

Consumer acceptance presents another point of contention. Cultural identity, culinary memory, and resistance to lab-derived ingredients continue to impede the widespread adoption of alternative proteins. As has been shown, public acceptance depends as much on cultural narrative integration as on sustainability indicators or health claims, but also on the successful integration of these innovations into culturally resonant culinary logics (19, 20). Consequently, the fifth paradigm, while theoretically compelling, continues to present itself unevenly in the realms of production and reception.

Noma's trajectory exemplifies these unresolved tensions. While emblematic of the innovation developed in the fourth paradigm, its founders eventually acknowledged the structural unsustainability of haute cuisine, citing the difficulty of maintaining trained kitchen teams, their high economic cost, the dependence of these restaurants on economic elites, and their difficulty integrating into the cultural hegemony of the moment. Its closure and subsequent reinvention as a research platform (Noma Projects) do not signal a failure, but rather a paradigmatic reorientation. As Mejía and Wilson (83) argue, Noma's evolution illustrates the need for epistemic humility and structural reform in haute cuisine before integrating into the future, marking a shift away from extractive models of excellence toward a postdisciplinary, regenerative ethic of practice.

While elitism, symbolic commodification, and technosolutionism remain real risks within this paradigm, several strategies have emerged to counterbalance these tendencies. Some projects seek to decenter the locus of authorship—through community partnerships, indigenous knowledge networks, or the rejection of aesthetic overproduction. Others aim to democratize access to epistemic innovation, not by diluting it, but by rooting it in shared practices and local ecologies. The much-discussed reinvention of Noma, including its announced closure as a restaurant and its pivot toward research and education, may be read as an attempt to escape the logic of perpetual exclusivity. Similarly, initiatives such as Midunu and Native, despite their modest scale, illustrate how post-digital culinary values can be adapted to regional contexts without sacrificing philosophical depth. The progressive transformation of Aponiente—where a former emblem of marine haute cuisine is now exploring low-value species, discarded sea biomass, and ecological regeneration—offers a powerful example of this shift. These cases suggest that the Fifth Paradigm is not blind to its contradictions but is evolving through self-critique, structural experimentation, and efforts to align ambition with inclusivity.

### 4.2 Hybrid knowledge systems and epistemological realignment

A defining feature of the fifth gastronomic paradigm is the hybridization of distinct knowledge systems, which dissolves the rigid boundaries between chef, scientist, artist, designer, and activist. Culinary research and development now take place in interdisciplinary ecosystems that incorporate microbiology, sensory neuroscience, sustainability science, anthropology, and digital technologies. As evidenced by interdisciplinary gastronomic R&D teams, these collaborations foster innovation by connecting experimental cuisine with scientific and cultural research (4, 64). This epistemological shift has been framed as a transition from gastronomy to “gastrology,” a complex, plural, and reflexive knowledge system (67). Moreover, some approaches extend to human-nonhuman interactions, rethinking culinary creativity through microbial intervention and technological mediation (66). Mugaritz's collaborative model exemplifies this convergence, where microbial fermentation, philosophical experimentation, and performative narrative intertwine into a unified epistemic project. As Galarraga and Albeniz describe, these interdisciplinary networks displace the solitary genius model and reframe gastronomic innovation as a systemic, interdisciplinary, multi-territorial process grounded in sustainability and ecology (65).

In this context, epistemic legitimacy is no longer based solely on institutional authority or expert accreditation. As Perullo argues, the democratization of taste—through digital platforms such as Instagram, TripAdvisor, or independent food blogs—has eroded the vertical hierarchies previously championed by Michelin or Gault and Millau (3). While this diffusion may challenge the coherence of gastronomic discourse, it simultaneously opens space for diverse epistemologies, including Indigenous, feminist, and multispecies forms of knowledge. The chef, in this model, becomes an epistemic mediator who translates between symbolic universes, ecological urgencies, and embodied knowledge (94).

Contemporary consumer behavior supports this epistemological shift. Studies show that today's diners increasingly value harmony, narrative authenticity, and cultural coherence over theatrical novelty or visual excess (95). Aesthetic innovation is now measured less by surprise or provocation than by its capacity to generate emotional, cognitive, and ecological resonance (28, 65). This aligns with the fifth paradigm's emphasis on the integrity between content, ethics, and sensorial form.

Digital and post-digital environments further contribute to this realignment. As explored, immersive gastronomic spaces and augmented culinary interfaces dissolve static food as an object of consumption, reconfiguring it as a site of participatory knowledge creation (38, 40, 80). In these environments, food becomes a medium through which memory, data, territory, and multisensory design converge. In these settings, food becomes a medium through which cultural memory, data infrastructures, territorial belonging, and multisensory design converge. Gastronomic innovation increasingly emerges from hybrid ecosystems that combine technological infrastructures, cultural narratives, and sensory strategies aligned with sustainability (65). This post-disciplinary ethos fosters a gastronomy that is not only experiential, but epistemologically generative—bridging affect, science, and ritual to cultivate new grammars of food rooted in ecological intelligence (94, 96).

The recognition of multispecies agency within the Fifth Paradigm is not simply metaphorical or symbolic. Rather, it implies a shift in the ontological status of non-human participants—from raw materials to epistemic collaborators. Fermenting microbes, fungal cultures, and living environments are no longer passive instruments but co-authors of culinary outcomes. This reconfiguration displaces the figure of the chef as sole sovereign creator and replaces it with a model of distributed authorship, where control is shared with systems that exceed human intentionality (3, 89). Ethically, this calls for a move beyond species extractivism toward multispecies solidarity, where the integrity and autonomy of microbial and ecological agents are acknowledged. Epistemologically, it positions gastronomy as a field where knowledge emerges from negotiated interactions between species, technologies, and ecologies—rather than being imposed from above. This view has practical consequences, as it requires chefs and researchers to attend to microbial needs, temporalities, and environmental thresholds as part of the creative process (33).

### 4.3 Food as ontological and political act

Among the most radical implications of the fifth gastronomic paradigm is its ontological repositioning of cooking—not as an artisanal technique, but as a site of ecological entanglement and epistemic redefinition. Gastronomy is no longer considered simply a vehicle for cultural expression or aesthetic performance, but a systemic and transformative act that reconfigures human-nonhuman relations, environmental trajectories, and epistemic justice. Loring's (2022) concept of “change conservation” usefully reframes food systems not as static infrastructures, but as generative interfaces that sustain feedback across human and ecological timescales (33).

This ecological reconceptualization is deepened by the rise of a posthumanist gastronomic discourse. Within this framework, regenerationist logics converge with multispecies agencies: microbes, soil ecologies, fungi, and artificial intelligence systems collaboratively redefine culinary creation as a distributed, more-than-human task. This shift destabilizes the anthropocentric myth of authorship, in which the chef appears as the sovereign orchestrator of meaning and matter. From this perspective, fermentation, foraging, and the recovery of ancestral techniques are not nostalgic reenactments, but ontological interventions—practices that instantiate alternative modes of relationality, sensory knowledge, and cohabitation. They reconfigure what it means to know, to cook, and to be in relation. As Perullo (2023) proposes, gastronomy must begin to recognize the material intertwinings and multispecies solidarities that constitute contemporary food systems (3).

However, these transformations are not equitably distributed. Access to the cultural, technological, and financial resources necessary to participate in the fifth paradigm remains deeply unequal. As Bowen et al. (97) argue, the most ethically and ecologically ambitious forms of gastronomy are often accessible only to elites, even when these rhetorically embrace inclusivity. If the fifth paradigm aspires to systemic transformation, it must confront these contradictions and articulate models based on inclusion, affordability, and socio-territorial justice. Equally important is the symbolic power of food. As Johnston and Baumann (98) remind us, smell and taste remain powerful senses where social distinction is both recognized and contested. In this sense, gastronomic innovation is never ideologically neutral: it is rooted in cultural negotiations about identity, memory, class, and power. The future of food, therefore, hinges not only on technological breakthroughs, but on the ethical and political reinvention of its roles—as sustenance, as symbolic terrain, and as an active strategy for multispecies coexistence and collective survival (99).

While the case studies discussed throughout this article are drawn primarily from the domain of haute cuisine, this should not imply that the paradigm is restricted to elite contexts. On the contrary, the systemic and regenerative logic that defines Post-Digital Integrative Cuisine holds significant potential for broader food systems—including mass catering infrastructures, traditional territorial cuisines, and community-based kitchens. Moreover, through imitation, appropriation, or osmotic diffusion, many of the experimental practices pioneered in avant-garde restaurants eventually permeate mainstream culinary culture (98). This influence can be seen in both large-scale restaurant chains and small, independent establishments that gradually adopt techniques, aesthetics, or narratives originating from fine dining. The paradigm's principles may thus inform new directions in public food programs, hospital nutrition, or agroecological policies grounded in local knowledge. The extension of these principles to national or institutional food strategies remains an important frontier for future research. However, the relative opacity of public policy frameworks and the difficulty of accessing consistent national-level data currently limit a more detailed analysis within this article (33).

### 4.4 Anticipatory imagination and the design of regenerative futures

A defining contribution of the fifth gastronomic paradigm is its anticipatory imagination: the capacity to conceive, simulate, and prototype futures that are ecologically viable, culturally plural, and ethically grounded. In this context, gastronomy is no longer a reactive practice that adapts to socio-environmental crises, but rather a prefigurative force that helps design post-extractive, multispecies, and post-disciplinary futures.

This anticipatory logic is visible in initiatives that explicitly align culinary creativity with systemic foresight. Projects such as Noma Projects and the now-defunct Space10 (Denmark) demonstrate how gastronomic institutions can evolve into speculative research platforms (82–84). At Noma Projects, the focus has shifted to fermentation, long-term food preservation, and climate-adapted ingredients, all informed by cross-sectoral collaborations with biologists, designers, and philosophers (83). These efforts reconfigure food not merely as sustenance or cultural expression, but as a speculative medium for prototyping futures through sensorial and symbolic practice.

From a design perspective, the fifth paradigm encourages a redefinition of culinary aesthetics toward what might be called regenerative design: the use of biotechnological tools, local ecologies, and multispecies logics to create dishes that restore rather than deplete. Examples include the cultivation of edible algae for urban environments (57), the integration of insect proteins into familiar culinary formats (100), or the exploration of lab-grown meat alternatives (16) with narrative and symbolic resonance. Chefs and food designers, in this model, are not just artisans but also future-makers who prototype possible worlds through the plate.

Anticipatory imagination also entails socio-political stakes: the construction of food futures is inseparable from justice, access, and sovereignty. As has been argued “civilizational change requires not only technological innovations but also new cosmovisions”; gastronomy, as a symbolic and sensorial system, is particularly suited to embody and disseminate those alternative visions (99). Culinary experiences that integrate climate literacy, indigenous cosmologies, or speculative storytelling can foster forms of experiential futuring that challenge dominant temporalities of consumerism and disposability (101).

In this sense, food becomes a medium of temporal disruption, allowing diners and creators alike to rehearse ecologies of care, degrowth imaginaries, or post-humanist ethics. This is already visible in restaurants like Boragó (Chile), Alchemist (Denmark), or Native (Singapore), where foraging, fermentation, microbial agency, and multispecies design are not gimmicks but epistemological strategies.

The intersection between artificial intelligence and gastronomy also offers fertile ground for anticipatory experimentation. Algorithmic tools are increasingly being used to generate novel recipes, simulate food aesthetics, optimize nutritional profiles, or improve training environments in culinary schools (29, 30). However, within the fifth paradigm, the goal is not techno-solutionism but symbiotic intelligence: a human–machine collaboration that respects ecological limits, aesthetic depth, and cultural complexity. As such, AI can become a co-designer of regenerative cuisines when situated within ethical and epistemological constraints aligned with long-term planetary viability (31, 32).

In contrast to the closed cycles of elitist haute cuisine, this paradigm calls for public imagination, plural participation, and the development of transdisciplinary food labs that link culinary art with the tools of futures studies, environmental humanities, and regenerative science (101).

Through this lens, gastronomy is no longer a mirror of the world as it is but a speculative sketchbook for reimagining how we might eat, dwell, and coexist in regenerative worlds yet to come. The fifth paradigm thus demands not only epistemic humility and ethical coherence but also visionary courage: the willingness to cook, serve, and share the future before it arrives.

While this paradigm emphasizes speculative foresight and systemic transformation, its long-term viability ultimately depends on embodied multisensory engagement. Human responses to taste, smell, texture, and visual form remain decisive forces in food perception, choice, and satisfaction (42). As such, anticipatory imagination must be grounded not only in ecological vision or technological simulation, but also in an affective and neurobiological understanding of how food is sensed and experienced (71). Sensory literacy and design, therefore, become integral to regenerative gastronomy—not as superficial styling, but as a material interface between proposed futures and embodied appetites (89). Projects that integrate neurogastronomy, immersive environments, and sensory mapping offer important clues for aligning future-oriented creativity with the persistent materiality of human taste and desire.

## 5 Conclusion

The emergence of the Post-Digital Integrative Cuisine represents an epistemological rupture in the history of gastronomy. Moving beyond codification, aesthetic provocation, or authorial centrality, this fifth paradigm redefines gastronomy as a regenerative, post-disciplinary practice that bridges ecology, ethics, and epistemology. Cooking is no longer merely a craft, but a speculative, multispecies act of world-making.

This paradigm embeds collective intelligence, biotechnological co-creation, indigenous knowledge, and sensory experimentation into a holistic system. Here, microbial cultures, AI, and ancestral techniques converge to articulate new culinary grammars rooted in ecological intelligence and planetary care. Far from being speculative, this model is already visible in projects like Mugaritz, Atelier Crenn, Owamni, or Alchemist, where gastronomy functions as a cultural interface for anticipatory, ethical, and transdisciplinary futures.

Yet tensions remain. Between innovation and access, spectacle and justice, the fifth paradigm must navigate contradictions inherited from previous models. The consolidation of this transformation depends on gastronomy's ability to serve as a space of epistemic repair and socio-ecological imagination.

The Post-Digital Integrative Cuisine is not an abstract ideal, but an emergent practice. It challenges chefs, researchers, and communities to reimagine food as a medium of multispecies solidarity, systemic resilience, and situated knowledge. To cook the future is not only to feed, but to care, to question, and to co-create new worlds at the table.

## Data Availability

The original contributions presented in the study are included in the article/supplementary material, further inquiries can be directed to the corresponding author.
